# Synthesis and Application of a pH-Responsive Functional Metal–Organic Framework: In Vitro Investigation for Delivery of Oridonin in Cancer Therapy

**DOI:** 10.3390/molecules29112643

**Published:** 2024-06-04

**Authors:** Jingyi Shen, Fangxin Gao, Qian Pan, Zhihui Zong, Lili Liang

**Affiliations:** Department of Pharmaceutical Engineering, Anhui Province Key Laboratory of Translational Cancer Research, Bengbu Medical University, Donghai Avenue, Bengbu 233030, China; 17555279938@163.com (F.G.); 15178350475@163.com (Q.P.); 0200205@bbmc.edu.cn (Z.Z.)

**Keywords:** zeolitic imidazolate framework-8, oridonin, cytotoxicity, nanocarriers, drug delivery

## Abstract

Oridonin (Ori) is a naturally existing diterpenoid substance that mainly exists in the Chinese medicinal plant Rabdosia rubescens. It was previously found to possess intriguing biological properties; however, the quick clearance from plasma and limited solubility in water restricts its use as a drug. Several metal–organic frameworks (MOFs), having big surfaces and large pores, have recently been considered promising drug transporters. The zeolitic imidazolate framework-8 (ZIF-8), a form of MOF consisting of 2-methylimidazole with zinc ions, is structurally stable under physiologically neutral conditions, while it can degrade at low pH values such as in tumor cells. Herein, a nanosized drug delivery system, Ori@ZIF-8, was successfully designed for encapsulating and transporting oridonin to the tumor site. The drug loading of the prepared Ori@ZIF-8 was 26.78%, and the particles’ mean size was 240.5 nm. In vitro, the release of Ori@ZIF-8 exhibited acid sensitivity, with a slow release under neutral conditions and rapid release of the drug under weakly acidic conditions. According to the in vitro anti-tumor experiments, Ori@ZIF-8 produced higher cytotoxicity than free Ori and induced apoptosis in A549 cancer cells. In conclusion, Ori@ZIF-8 could be a potential pH-responsive carrier to accurately release more oridonins at the tumor site.

## 1. Introduction

The diterpenoid compound Oridonin (Ori), which is extracted from a conventional Chinese herb called Rabdosia rubescens [1], has been proven to possess notable therapeutic properties, such as anticancer [2], anti-inflammation [3], anti-depression [4], neuroprotection [5], anti-angiogenic [6] and antiviral [7] activities. Its inhibitory benefits against many malignant tumors have been confirmed, especially breast cancer [8], gastric cancer [9], leukemia [10], lung cancer [11,12], esophageal cancer [13], and liver cancer [14,15]. However, Ori’s therapeutic usage for anticancer purposes is severely constrained because of its poor bioavailability, limited in vivo half-life, and inadequate solubility in water [16,17,18]. Thus, the development of an effective transport system is essential to increase the drug’s half-life and in vivo bioavailability. In recent years, many technologies, like cyclodextrin-inclusion complexes [19], microparticles [20], and liposomes [21], have been used for loading Ori, and these systems may increase the solubility and alter the in vivo process. However, these preparations also have some defects, such as low stability, high toxicity, and limited specificity [22]. In the meantime, nanotechnology has developed rapidly in the pharmaceutical field. Compared with direct drug administration, nanoscale preparations have the advantages of a long cycle time, improved pharmacokinetics, and small toxic side effects [23,24,25]. In addition, nanocarriers can escape rapid drug clearance by the mesh endothelial system and improve drug efficacy [26,27].

Metal–organic frame materials (MOFs) are a type of 3D-ordered polyporous hybrid material that is generated by combining metal ions with organic molecules via ligand bonds [28]. The adjustable composition and topology, highly ordered pores, large surface area, and excellent physical and chemical properties [29,30,31] give MOFs great potential in the biomedicine field. Zeolitic imidazolate framework material (ZIFs) is a MOF material with a zeolite topology synthesized from metal ions such as Zn^2+^ or Co^2+^ and ligands of imidazole or its derivatives [32]. As a subclass of MOFs, this material has excellent characteristics, including having a huge surface area, numerous pores, high thermal resistance, and inherent biodegradability [33,34,35]. Based on this, ZIFs are good candidates in the area of biomedicine, including the delivery of drugs and the treatment of cancers. ZIF-8, the most representative material of ZIFs material, is a three-dimensional tetrahedral structure formed by the zinc ions connected to the N atom in the 2-methylimidazole (2-MIM) [36]. In addition to the traditional advantages of ZIFs, improved structural stability, better biological compatibility, and enhanced capability for drug loading are further advantages of ZIF-8 [37,38,39]. In particular, the ZIF-8 material also has excellent pH sensitivity, which dissolves in the weak acid cell milieu of tumorous tissues, but it maintains its structural stability during neutral circumstances [40,41,42]. This feature enables the controllable and accurate release of loaded drugs at the location of tumors. These advantages allow ZIF-8 a wide application in drug loading; ZIF-8 has become one of the most widely researched materials for MOFs.

Therefore, in this study, ZIFs-based pH-sensitive nanoparticles were designed and prepared for loading the small molecule Ori, aiming to increase Ori’s bioavailability and enhance its therapeutic properties. The physical and chemical characteristics of the prepared Ori@ZIF-8 were evaluated in terms of structure, morphology, and particle dimension. Its drug-loading capacity (DLC) and release properties of Ori, in vitro, were measured by ultraviolet–visible spectrometry. Moreover, the anticancer effect and mechanism on cancer cells of Ori@ZIF-8 were also investigated by cell cytotoxicity testing and cell apoptosis analysis.

## 2. Results and Discussion

### 2.1. Preparation of Ori@ZIF-8

**Scheme 1:**
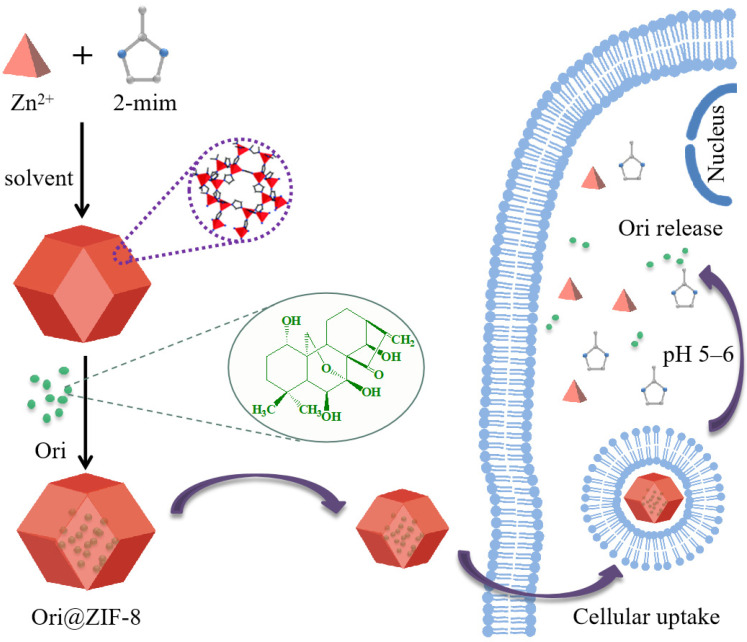
Schematic illustration of Ori@ZIF-8 construction and its drug release route.

The Ori@ZIF-8 drug delivery system, loaded with the small molecule Ori, was successfully one-pot prepared in this study. Briefly, a solution of 2-methylimidazole was stirred and mixed into a zinc nitrate methanol-aqueous solution containing Ori; the reaction was kept at room temperature for 2 h, and then Ori@ZIF-8 was obtained by centrifugal separation and purified by washing. Meanwhile, free ZIF-8 was prepared by the same operation as the control. The preparation process of Ori@ZIF-8 is illustrated as Scheme 1.

### 2.2. Morphologies and Particle Size

**Figure 1:**
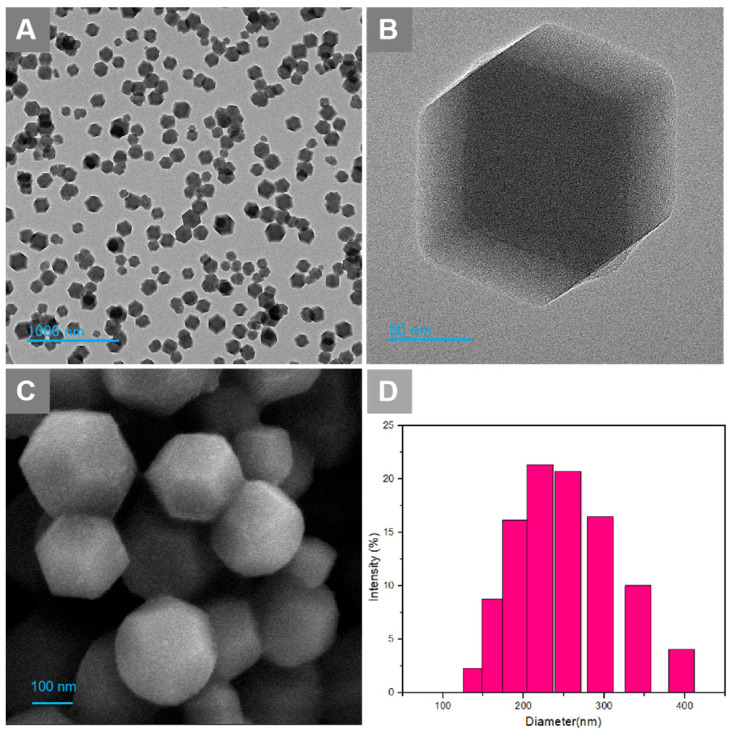
(**A**) TEM image of Ori@ZIF-8 NPs; (**B**) TEM image of a single nanosphere; (**C**) SEM image of Ori@ZIF-8 NPs; (**D**) the particle size distributions of Ori@ZIF-8.

The obtained Ori@ZIF-8 nanoparticles were characterized morphologically via SEM and TEM, while the particle size dispersion was measured using dynamic light scattering (DLS). The Ori@ZIF-8 nanoparticles dispersed and exhibited a uniform, fine, regular sphere-like dodecahedron morphology (Figure 1A–C). The size range for Ori@ZIF-8, shown in Figure 1D, indicated that the mean size of Ori@ZIF-8 is 240.5 nm (PDI = 0.155), with a narrow distribution, presenting a relatively uniform particle size distribution range. These outcomes showed that Ori@ZIF-8 nanoparticles with a good shape and uniform size were successfully produced.

### 2.3. PXRD and FTIR Analysis

**Figure 2:**
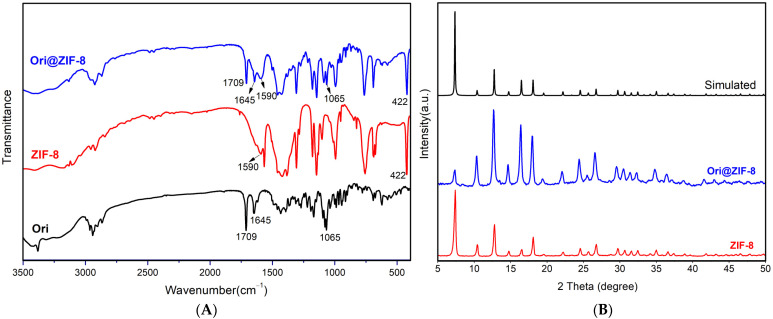
The FTIR spectra (**A**) of Ori@ZIF-8, ZIF-8, and Ori; PXRD patterns (**B**) of Ori@ZIF-8, ZIF-8, and simulated ZIF-8 (CCDC 602542).

Fourier infrared spectroscopy (FTIR) was used to investigate the chemical bond vibrational and stretching peaks in the structures of ZIF-8, Ori@ZIF-8, and Ori (Figure 2A).

In the profiles of Ori@ZIF-8 and ZIF-8, the stretching vibration of the nitrogen heterocycle’s C=N bond occurred at 1590 cm^−1^ and with a Zn-N bond at 422 cm^−1^, which demonstrated the effective preparation for ZIF-8 [43]. Infrared spectra of Ori@ZIF-8 and Ori both showed absorption peaks at 1709 cm^−1^ (bands corresponding to the stretching vibration of the C=O bond), 1645 cm^−1^ (C=C bond), and 1065 cm^−1^ (C-O-C bond) [44], confirming that oridonin was present in Ori@ZIF-8’s structure. Therefore, it was verified that the Ori@ZIF-8 nanoparticles could be prepared with success.

To further determine the success of Ori@ZIF-8’s preparation, the crystal structures of ZIF-8 and Ori@ZIF-8 were characterized by PXRD. As shown in Figure 2B, the PXRD crystal data of the synthesized ZIF-8 were identical to the data provided in the previous literature (Simulated in Figure 2B) [45], and ZIF-8’s effective synthesis was demonstrated. The PXRD for Ori@ZIF-8 was consistent with ZIF-8 and the simulated ZIF-8. This result demonstrated that the crystal structures of Ori@ZIF-8 and ZIF-8 were similar, showing that with the loading of oridonin, ZIF-8’s crystal structure remained unaltered.

The stability of Ori@ZIF-8 was conducted via PXRD. Ori@ZIF-8 NPs were placed in PBS with FBS (10%) over different times and then the PXRD patterns were obtained (see Figure S1, Supporting Information). All tested Ori@ZIF-8s could maintain their crystal structures well, indicating that these NPs showed good stability under physiological conditions.

### 2.4. Thermogravimetric (TG) Analyses

**Figure 3:**
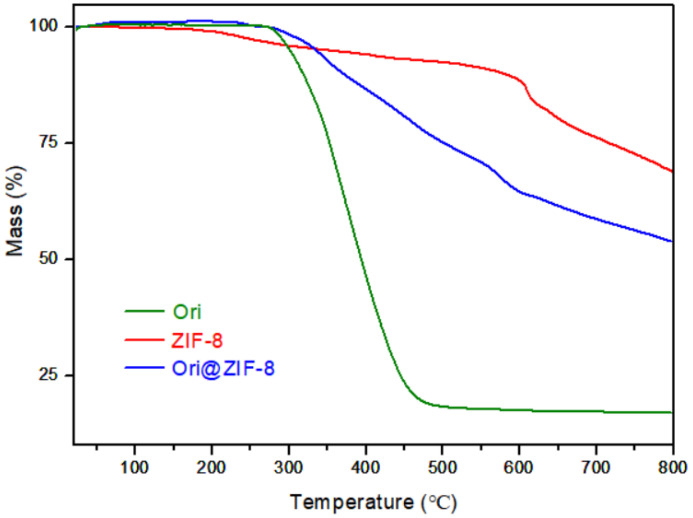
TG analysis curves of Ori, ZIF-8, and Ori@ZIF-8.

Thermogravimetric (TG) analyses of Ori@ZIF-8, ZIF-8, and Ori were performed to study their thermal effects. As shown in Figure 3, Ori@ZIF-8 was relatively stable below 300 °C, while ZIF-8 had a slight loss of weight. This difference may be caused by the residual guest molecules in the blank ZIF-8, such as the organic ligands or residual solvent. Ori@ZIF-8 had about a 25% weight loss at 300 °C to 500 °C, possibly resulting from the breakdown of the drug oridonin. The weight loss above 500 °C was most likely caused by the structural collapse of the ZIF-8 frame [46].

As a supplementary measure, N_2_ adsorption and desorption analyses were carried out (see Figure S2, Supporting Information). The BET surface area and pore size of ZIF-8 were measured to be 1652.6 m^2^/g and 1.52 nm, respectively, which facilitated the encapsulation of Ori in ZIF-8.

### 2.5. Drug-Loading Capacity and In Vitro Drug Release

As important parameters, the drug-loading capacity and encapsulation rates can be determined and calculated by UV–visible spectrophotometry (see Figures S3 and S4, Supporting Information). The drug-loading and encapsulation rates were calculated to be 26.78 ± 0.55% and 56.11 ± 0.65%, respectively. This result could be generally consistent with the TG analysis of Ori@ZIF-8. Furthermore, CNH elemental analysis data for ZIF-8 and Ori@ZIF-8 (see Table S1, Supporting Information) also supported the content of Ori in Ori@ZIF-8 nanoparticles.

**Figure 4:**
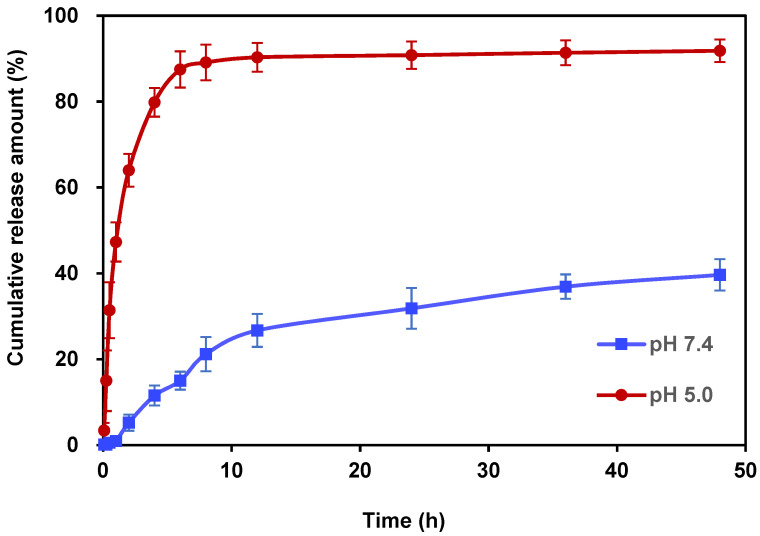
In vitro drug release profile for Ori@ZIF-8 at pHs 7.4 and 5.0 (*n* = 3).

For the in vitro release behavior studies, PBS with pH values of 7.4 and 5.0 was used as the drug release medium to verify the acid responsiveness of Ori@ZIF-8 (see Figures S5 and S6, Supporting Information). The pH 7.4 and 5.0 PBS solutions simulated the healthy physiological conditions of humans and the acidic environment found in tumor cells, respectively. Figure 4 depicts the Ori in vitro release profile of Ori@ZIF-8 nanoparticles. The release of oridonin in Ori@ZIF-8 was relatively fast under the condition of pH 5.0, which showed a rapid growth trend, with a cumulative release of 79.81% in the first 4 h. However, throughout that same time frame, only 11.55% of the medication was released at pH 7.4. After 48 h, 91.83% of Ori was cumulatively released in PBS at pH 5.0, whereas at pH 7.4, just 39.56% of Ori was released. The acidic condition clearly promoted the drug release behavior of Ori@ZIF-8, which might be attributed to the responsive degradation of ZIF-8 [47]. Therefore, the obtained Ori@ZIF-8 showed good pH responsiveness. This property facilitates the release of Ori in the acidic environment of tumor tissues while maintaining a stable structure under neutral conditions.

### 2.6. In Vitro Anti-Tumor Activity

**Figure 5:**
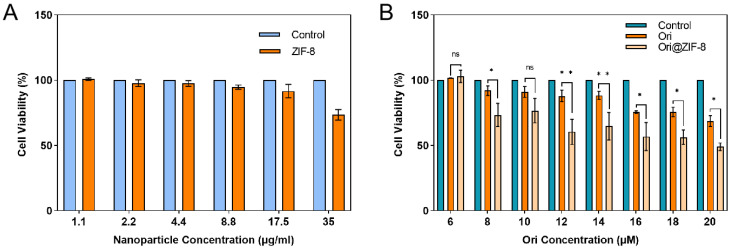
Viabilities of A549 cells cultured with blank ZIF-8 NPs (**A**) and different concentrations of drugs (**B**) (*n* = 3); ns for not significant, * *p* < 0.05, ** *p* < 0.01.

The cytotoxicity of Ori@ZIF-8 was evaluated. Human lung cancer A549 cells were cultured for 24 h with varying drug doses, and then CCK-8 was used to assess the cell viability results. Normal-growing tumor cells without drug treatment were used as a control. Figure 5 illustrates that the A549 cell survival rate was not significantly reduced after incubation for 24 h with blank ZIF-8 NPs in a concentration range of 0 to 35 μg/mL, which indicated that the ZIF-8 NPs had no obvious cytotoxicity and exhibited good biocompatibility within a certain concentration range. In contrast, both free Ori and the Ori@ZIF-8 NPs showed significant inhibition to A549 cells, and the inhibitory effect showed a certain dose dependence. Ori@ZIF-8 NPs exhibited a better inhibitory effect in A549 cells than free Ori under equal Ori concentrations, demonstrating that Ori@ZIF-8 could effectively load Ori to achieve a therapeutic effect.

Meanwhile, the biologic compatibility of blank ZIF-8 and Ori@ZIF-8 was evaluated using 293T human embryonic kidney cells using a CCK-8 assay. The results indicated that ZIF-8 NPs showed no significant cytotoxicity for 293T cells (see Figure S7A, Supporting Information). Meanwhile, the cytotoxicity of both free Ori and Ori@ZIF-8 against the 293T cells showed some concentration dependence. At the same Ori concentration, the cytotoxicity of free Ori on 293T cells was greater than that of Ori@ZIF-8, suggesting that Ori loading reduced the cytotoxicity of the drug and improved biocompatibility (see Figure S7B, Supporting Information).

### 2.7. Cell Apoptosis

**Figure 6:**
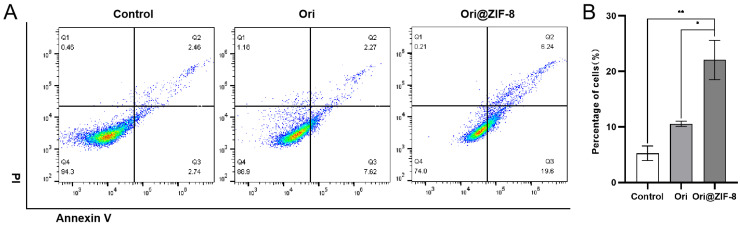
Results of apoptosis of A549 cells. (**A**) Flow cytometry analysis after treatment with Ori and Ori@ZIF-8; (**B**) statistical analysis results of apoptosis in cells, * *p* < 0.05, ** *p* < 0.01.

Apoptosis experiments were conducted by flow cytometry to quantitatively investigate the apoptotic effect of our prepared nanoparticles. A549 cancer cells were treated with Ori or Ori@ZIF-8 to determine the percentages of cell apoptosis following a 24 h incubation period. According to Figure 6, the percentage of cell apoptosis in the control group was 5.20%, and the percentage in the free Ori group was 9.89%. The apoptosis rate of the Ori@ZIF-8 group was higher than the free Ori group (*p* < 0.05), and the average apoptosis rate was 25.84% at the same Ori concentration. It was speculated that the Ori@ZIF-8 nanoparticles were uptaken into tumor cells through endocytosis and subsequently released Ori under the acidic conditions of the tumor, ultimately promoting cell apoptosis.

In summary, uniform and stable Ori@ZIF-8 nanoparticles were successfully prepared by a one-pot method, and PXRD, SEM, and TEM proved the purity, morphology, and crystalline shape of the Ori@ZIF-8 NPs. The prepared Ori@ZIF-8 had a polyhedral shape with good crystallinity. FTIR, TG, UV–Vis, elemental analyses, and N_2_ adsorption and desorption analyses further proved the successful loading of Ori in the ZIF-8 nanoparticles and the drug-loading and encapsulation rates of Ori@ZIF-8, which were calculated to be 26.78 ± 0.55% and 56.11 ± 0.65%, respectively. The Ori@ZIF-8 nanoparticles had good stability in a neutral environment but showed a rapid drug release in an acidic environment. This reflects the fact that ZIF-8 has the additional advantages of high drug-loading efficiency and pH-responsiveness in comparison with other MOFs. Viabilities of free Ori, ZIF-8, or Ori@ZIF-8 NPs to normal cells and tumor cells cultured showed that the prepared ZIF-8 NPs have good biocompatibility and can be used as relatively safe drug carriers. Ori@ZIF-8 NPs exhibited a better inhibitory effect on the A549 cells than free Ori. This enhanced cytotoxicity may be caused by the effective endocytosis of NPs by tumor cells.

## 3. Experimental Section

### 3.1. Materials

Oridonin (99%) was provided by Shanghai Shenghong Bio-Tech Co. (Shanghai, China), 2-methylimidazole (99%) was obtained from Shanghai Macklin Biochemical Technology Co. (Shanghai, China), and Zn(NO_3_)_2_·6H_2_O (99%) was acquired from Shandong Xiya Chemical Co. (Linyi, China). The remaining chemicals and solvents were all of the analytical reagent class (AR).

### 3.2. Procedure for Ori@ZIF-8 Preparation

The drug oridonin was effectively encapsulated to create the Ori@ZIF-8 carrier by a one-step method. Zn(NO_3_)_2_·6H_2_O (150 mg) and oridonin (60 mg) were dissolved in a methanol/distilled water solution (10 mL, 1:1), and methanol was added to increase the dissolution of oridonin. Then, 10 mL of the 2-methylimidazole aqueous solution (16.5 mg/mL) was stirred in while being added drop-by-drop to the solution above. At room temperature, this system was stirred continuously for two hours. After that, the white precipitate was obtained after centrifuging for 10 min at 10,000 rpm and cleaned with methanol twice to remove unreacted reactants and other impurities; then, the Ori@ZIF-8 product was vacuum-dried.

The empty ZIF-8 was also produced using the same procedure as above for the control.

### 3.3. Material Characterization

The morphological properties of Ori@ZIF-8 were examined using scanning electron microscopy (SEM, Sigma 300-Smart EDX, ZEISS, Roedermark, Germany). Powder X-ray diffraction analysis (PXRD) was used to further characterize the structures of the synthesized ZIF-8 and Ori@ZIF-8. Powder X-ray diffraction analysis (PXRD, D8, Bruker, Karlsruhe, Germany) using Cu-K α radiation was used for further analyzing the structures of synthesized ZIF-8 and Ori-loaded nanoparticles with an angle range from 2θ = 3° to 2θ = 50°.

The particle size distribution of Ori@ZIF-8 nanoparticles was determined using a particle size potentiometer (ZS90, Malvern Panalytical Ltd., Malvern, UK). The samples were dispersed in a small amount of methanol and sonicated to obtain a favorable dispersion, and the assay was repeated three times.

The chemical structure of Ori-loaded ZIF-8 nanoparticles, ZIF-8 and Ori, was measured within the range of 3500 to 400 cm^−1^ using Fourier transform infrared spectroscopy (FTIR, NICOLET iS50, Thermo, Waltham, MA, USA). Thermogravimetric (TG) analyses of Ori-loaded ZIF-8 nanoparticles, ZIF-8, and Ori were evaluated on an STA 449-F5 analyzer (NETZSCH, Hanau, Germany) in a N_2_ environment within a thermal range of 25 °C to 800 °C at a temperature rise of 10 °C per minute. The measurement of the UV–visible spectrum using an Evolution 220 UV–Visible Spectrophotometer (Thermo, USA) was carried out to determine the drug content and the amount of Ori released from Ori@ZIF-8 in vitro.

### 3.4. Determination of Ori@ZIF-8’s Drug-Loading Capability

To ascertain the Ori content in the obtained Ori@ZIF-8, UV–visible spectrophotometry at room temperature was operated. For obtaining the calibration curve, a series of Ori–methanol solutions, with a concentration ranging from 0 to 50 μg·mL^−1^, were prepared. After weighing and dispersing 5 mg of Ori@ZIF-8 in 5 mL of methanol, 1 M hydrochloric acid was added to destroy the structure completely until the solution was clear and transparent [48]; then, methanol was added to 10 mL of the solution after it had been filtered using a microporous filter (0.22 μm). The absorbance of the above solution was detected at 238 nm by UV–vis and then calculated from the standard curve.

### 3.5. In Vitro Drug Release from Ori@ZIF-8

The assay of Ori, released by Ori@ZIF-8 in vitro, was carried out in three parallel groups. ZIF-8 and ORI@ZIF-8 were installed in dialysis bags (MWCO: 500 Da) and then added into phosphate-buffered saline (PBS, 100 mL) with pHs of 7.4 and 5.0, shaking at 37 °C, respectively. At predetermined intervals, 1 milliliter of releasing medium was precisely taken while 1 mL of fresh buffer was precisely supplemented simultaneously to maintain a constant volume (100 mL). The release medium was filtered through a disposable microporous filter membrane and the absorbance of the release medium at 238 nm was measured.

### 3.6. In Vitro Anti-Tumor Studies

The CCK-8 tests were used to evaluate the anti-tumor activities of Ori, ZIF-8, and Ori@ZIF-8 nanoparticles against A549 lung cancer cells, respectively. Cancer cells were grown in culture flasks containing the cell culture medium in an incubator with a CO_2_ concentration of 5% and a temperature of 37 °C. To achieve a cell concentration of 5000 cells for each well, 100 μL of the cancer cell solution was transferred to a 96-well plate and then incubated at 37 °C for 24 h. After the removal of the cell culture medium, fresh media containing different ratios of free Ori or Ori@ZIF-8 were used to treat the cells for 24 h. The CCK-8 test solution (10 μL) was applied to every well while the cells were cultured for 30 min at 37 °C, avoiding light. Utilizing an enzyme immunoassay, absorbances of each sample were assayed at 450 nm to assess the viability of cells.

### 3.7. Apoptosis

Apoptosis in the A549 cells was analyzed using flow cytometry after the treatment by Ori@ZIF-8 or free Ori. After being grown in plates with six wells at the appropriate density, the A549 cells were treated with either free Ori or Ori@ZIF-8 at the same Ori concentration, respectively. The treated cells were gained after a 24 h period and the binding agent was added; PI and AnnexinV-FITC were added whereafter and incubated at 25 °C for 15 min. The samples were examined by a flow cytometer to calculate the apoptosis rate.

## 4. Conclusions

With this work, we have effectively created a pH-sensitive delivery method for nanomedicines. A metal–organic frame material was selected as a drug carrier to wrap oridonin and transport it to the tumor site. The obtained Ori@ZIF-8 nanoparticles were confirmed to be structurally intact, with good morphology, low cytotoxicity, and excellent capability to load drugs. Drug release experiments of the nanoparticles in vitro exhibited an outstanding pH sensitivity, which could release the drug slowly under normal conditions and rapidly release the drug under acidic conditions. The in vitro cell experiments showed the tumor cytotoxicity of Ori@ZIF-8 nanoparticles. Moreover, Ori@ZIF-8 could induce apoptosis in A549 cells. These results all suggest that Ori@ZIF-8 can deliver Ori to A549 cancer cells and improve the cancer treatment efficacy. Therefore, the nanodrug loading system we prepared can be applied to cancer treatment.

## Data Availability

The data presented in this study are available on request from the corresponding author.

## Supplementary Materials

The following supporting information can be downloaded at: https://www.mdpi.com/article/10.3390/molecules29112643/s1, Figure S1: PXRD patterns of Ori@ZIF-8 NPs after placed in PBS with FBS (10%) over different times; Figure S2: Nitrogen adsorption/desorption isotherms of ZIF-8; Figure S3: UV spectra of Ori, ZIF-8, and Ori@ZIF-8 in methanol solution with hydrochloric acid; Figure S4: Calibration curve of Ori in methanol solution at 238 nm; Figure S5: Calibration curve of Ori at 238 nm in PBS buffer solution at pH 7.4; Figure S6: Calibration curve of Ori at 238 nm in PBS buffer solution at pH 5.0; Figure S7: Viabilities of 293T human embryonic kidney cells cultured with blank ZIF-8 NPs (A) and different concentrations of drugs (B) (*n* = 3); Table S1: The organic elemental analyses for Ori@ZIF-8 and ZIF-8 from EA.
