# Supplementary material for: Synthesis and Application of a pH-Responsive Functional Metal–Organic Framework: In Vitro Investigation for Delivery of Oridonin in Cancer Therapy
Source: Molecules. 2024 Jun 4;29(11):2643. doi: 10.3390/molecules29112643 (PMC11173415; doi:10.3390/molecules29112643)
Supplement: Supplementary file 1 [file molecules-29-02643-s001.zip › molecules-2966205-supplementary.pdf]

Synthesis and Application of a pH-Responsive Functional Metal–Organic Framework: In Vitro Investigation for Delivery of Oridonin in Cancer Therapy

Jingyi Shen *, Fangxin Gao, Qian Pan, Zhihui Zong and Lili Liang *

Department of Pharmaceutical Engineering, Anhui Province Key Laboratory of Translational Cancer Research, Bengbu Medical University, Donghai Avenue, Bengbu 233030, China; 17555279938@163.com (F.G.); 15178350475@163.com (Q.P.); 0200205@bbmc.edu.cn (Z.Z.)

* Correspondence: 2014003@bbmc.edu.cn (J.S.); 0200166@bbmc.edu.cn (L.L.)

Supporting Information

Figure S1. PXRD patterns of Ori@ZIF-8 NPs after placed in PBS with FBS (10%) over different times.

Stability experiments for Ori@ZIF-8 were conducted. Briefly, Ori@ZIF-8 NPs were placed in PBS with FBS (10%) over different times (2 days or 5 days), the crystal structure of Ori@ZIF-8 were characterized by PXRD after centrifugation and drying. As shown in Figure S1, all tested Ori@ZIF-8 NPs could maintain their crystal structures well, implying that these NPs showed good stability under physiological conditions.

Figure S2. Nitrogen adsorption/desorption isotherms of ZIF-8.

Figure S3. UV spectra of Ori, ZIF-8, and Ori@ZIF-8 in methanol solution with hydrochloric acid.

Figure S4. Calibration curve of Ori in methanol solution at 238 nm.

Figure S5. Calibration curve of Ori at 238 nm in PBS buffer solution at pH 7.4.

Figure S6. Calibration curve of Ori at 238 nm in PBS buffer solution at pH 5.0.

Figure S7. Viabilities of 293T human embryonic kidney cells cultured with blank ZIF-8 NPs (A) and different concentrations of drugs (B) (n = 3).

Table S1. The organic elemental analyses for Ori@ZIF-8 and ZIF-8 from EA.

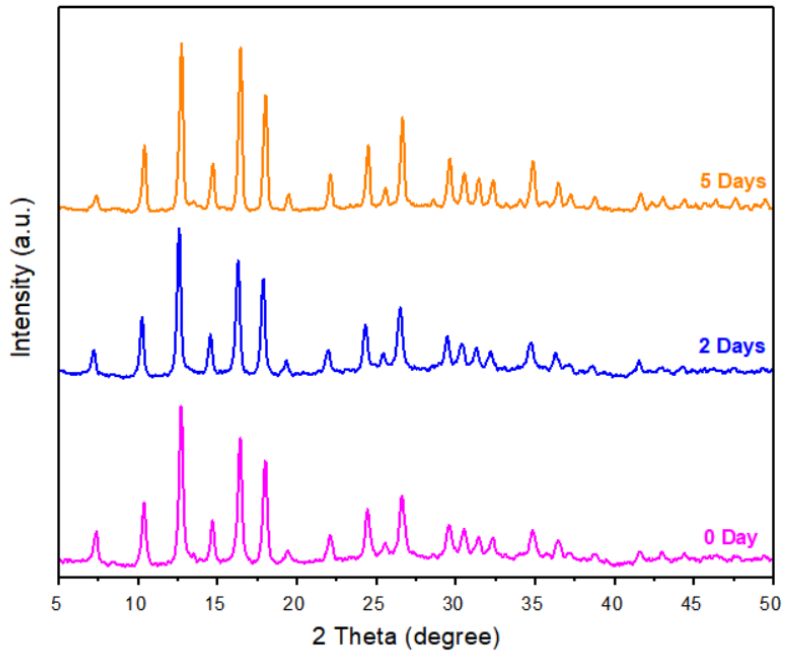

Figure S1. PXRD patterns of Ori@ZIF-8 NPs after placed in PBS with FBS (10%) over different times.

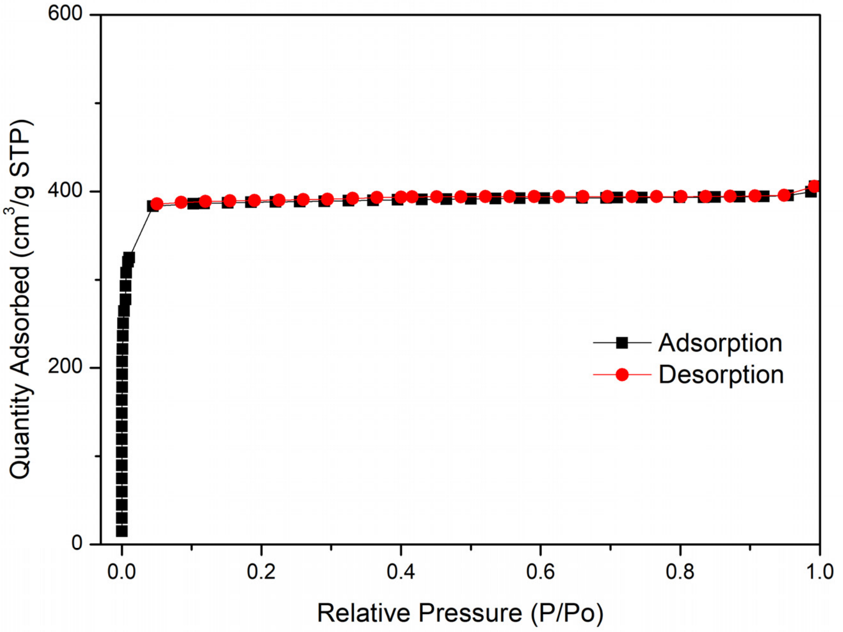

Figure S2. Nitrogen adsorption/desorption isotherms of ZIF-8.

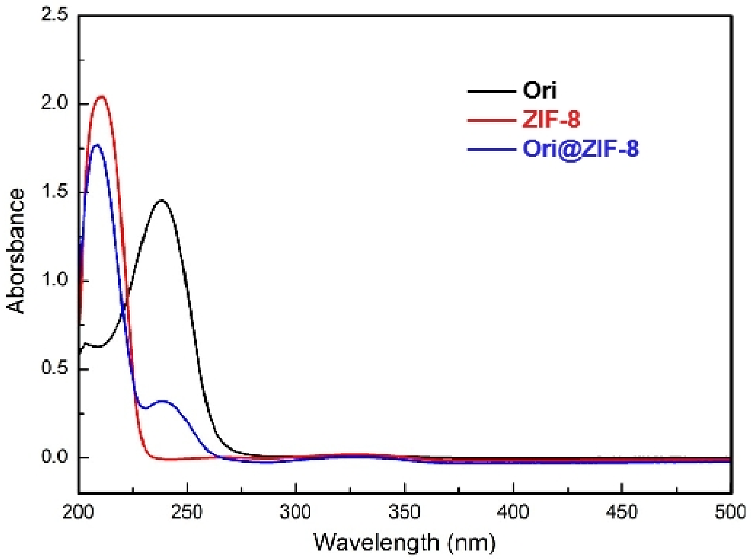

Figure S3. UV spectra of Ori, ZIF-8, and Ori@ZIF-8 in methanol solution with hydrochloric acid.

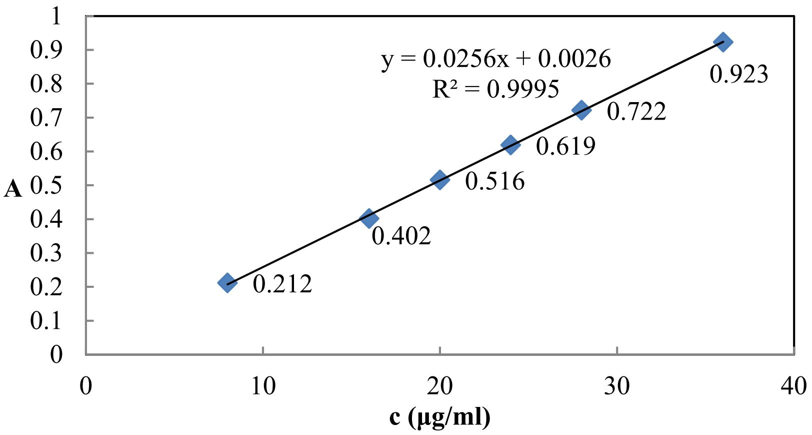

Figure S4. Calibration curve of Ori in methanol solution at 238 nm.

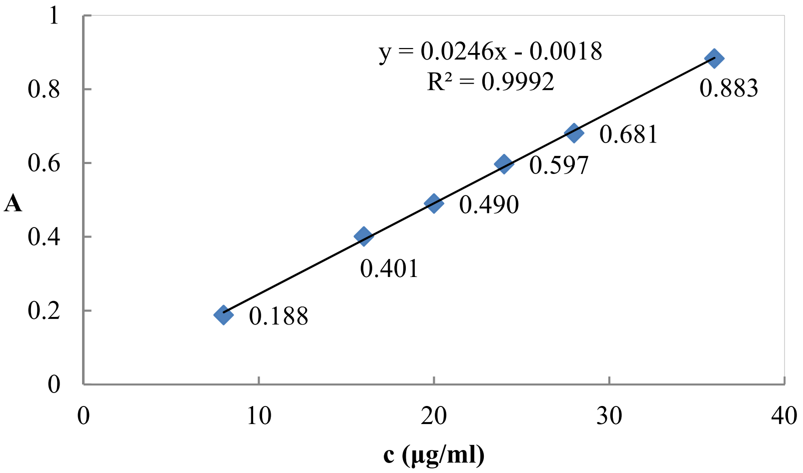

Figure S5. Calibration curve of Ori at 238 nm in PBS buffer solution at pH 7.4.

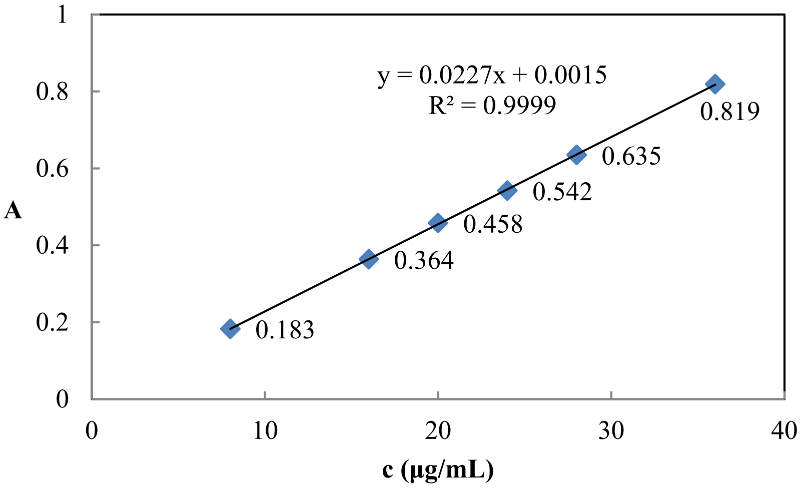

Figure S6. Calibration curve of Ori at 238 nm in PBS buffer solution at pH 5.0.

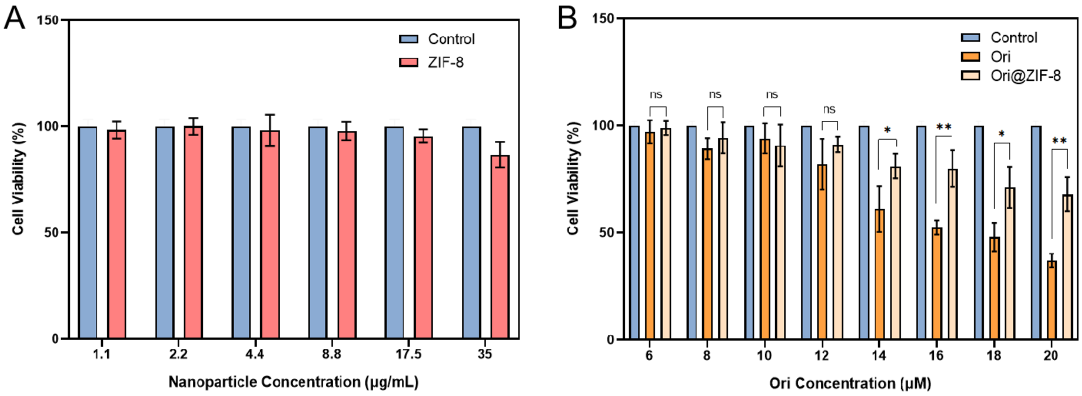

Figure S7. Viabilities of 293T human embryonic kidney cells cultured with blank ZIF-8 NPs (A) and different concentrations of drugs (B) (n = 3).

Table S1. The organic elemental analyses for Ori@ZIF-8 and ZIF-8 from EA.

Sample	Elements		
	N (%)	C (%)	H (%)
Ori@ZIF-8	15.97	48.96	5.61
ZIF-8	24.91	39.65	4.77
